# Transcriptomic Responses of *Fusarium verticillioides* to Lactam and Lactone Xenobiotics

**DOI:** 10.3389/ffunb.2022.923112

**Published:** 2022-06-20

**Authors:** Minglu Gao, Xi Gu, Timothy Satterlee, Mary V. Duke, Brian E. Scheffler, Scott E. Gold, Anthony E. Glenn

**Affiliations:** ^1^ Department of Plant Pathology, University of Georgia, Athens, GA, United States; ^2^ Institute of Bioinformatics, University of Georgia, Athens, GA, United States; ^3^ United States Department of Agriculture (USDA), Agricultural Research Service (ARS), U.S. National Poultry Research Center, Toxicology & Mycotoxin Research Unit, Athens, GA, United States; ^4^ United States Department of Agriculture (USDA), Agricultural Research Service, Genomics and Bioinformatics Research Unit, Stoneville, MS, United States

**Keywords:** RNA-Seq, BOA, 2-benzoxazolinone, 2-oxindole, 2-coumaranone, chlorzoxazone, gene clusters

## Abstract

The important cereal crops of maize, rye, and wheat constitutively produce precursors to 2-benzoxazolinone, a phytochemical having antifungal effects towards many *Fusarium* species. However, *Fusarium verticillioides* can tolerate 2-benzoxazolinone by converting it into non-toxic metabolites through the synergism of two previously identified gene clusters, *FDB1* and *FDB2*. Inspired by the induction of these two clusters upon exposure to 2-benzoxazolinone, RNA sequencing experiments were carried out by challenging *F. verticillioides* individually with 2-benzoxazolinone and three related chemical compounds, 2-oxindole, 2-coumaranone, and chlorzoxazone. These compounds all contain lactam and/or lactone moieties, and transcriptional analysis provided inferences regarding the degradation of such lactams and lactones. Besides induction of *FDB1* and *FDB2* gene clusters, four additional clusters were identified as induced by 2-benzoxazolinone exposure, including a cluster thought to be responsible for biosynthesis of pyridoxine (vitamin B6), a known antioxidant providing tolerance to reactive oxygen species. Three putative gene clusters were identified as induced by challenging *F. verticillioides* with 2-oxindole, two with 2-coumaranone, and two with chlorzoxazone. Interestingly, 2-benzoxazolinone and 2-oxindole each induced two specific gene clusters with similar composition of enzymatic functions. Exposure to 2-coumranone elicited the expression of the fusaric acid biosynthetic gene cluster. Another gene cluster that may encode enzymes responsible for degrading intermediate catabolic metabolites with carboxylic ester bonds was induced by 2-benzoxazolinone, 2-oxindole, and chlorzoxazone. Also, the induction of a dehalogenase encoding gene during chlorzoxazone exposure suggested its role in the removal of the chlorine atom. Together, this work identifies genes and putative gene clusters responsive to the 2-benzoxazolinone-like compounds with metabolic inferences. Potential targets for future functional analyses are discussed.

## Introduction

Plants have adopted various defensive mechanisms against microbial pathogens. One of the most notable mechanisms is the production of antimicrobial secondary metabolites. These phytochemicals may be constitutively produced by the healthy plant (phytoanticipins) or synthesized *de novo* when encountering pathogens (phytoalexins) (VanEtten et al., 1995). However, some plant pathogens are capable of detoxifying these bioactive phytochemicals, which complicates plant disease management and poses a threat to the health of agricultural crops (Osbourn, 1999).

As one of the phytoanticipins found in select members of the Poaceae, 2-benzoxazolinone (BOA) is known for its *in vitro* antagonistic effect against a wide range of microbes (Niemeyer, 1988; Glenn et al., 2001). BOA is the spontaneous degradation product of its unstable precursor, 2,4-dihydroxy-2*H*-1,4-benzoxazin-3(4*H*)-one (DIBOA), which is also a defensive compound known for inhibiting bacteria, fungi, and insect feeding (Corcuera et al., 1978; Woodward et al., 1978; Niemeyer, 1988; Hashimoto and Shudo, 1996; Bravo et al., 1997; Glenn et al., 2001).

**Figure 1:**
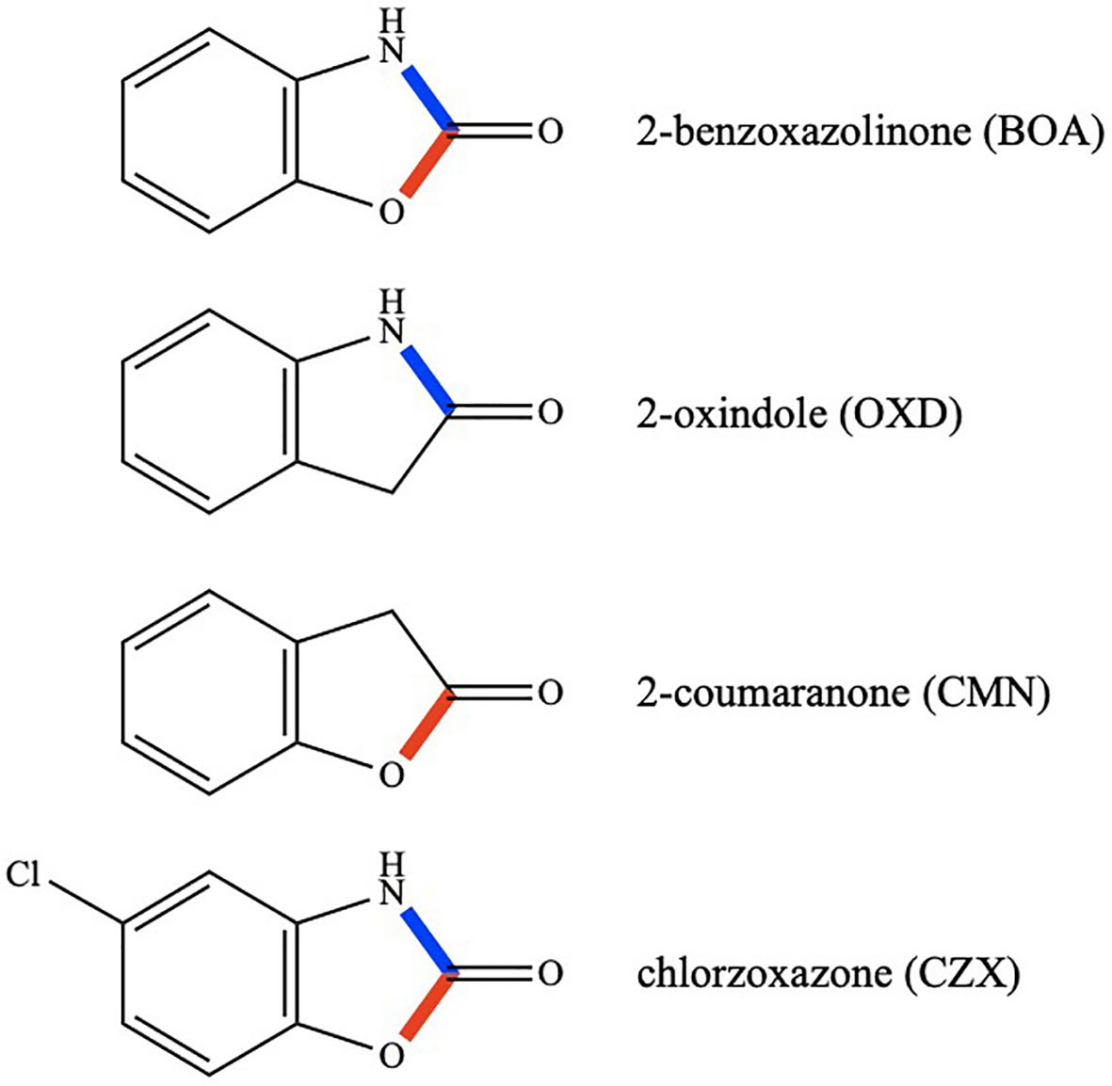
Chemical structures of BOA, OXD, CMN, and CZX. Lactam bonds are shown in blue and lactone bonds in red.

*Fusarium verticillioides* is one of the most prevalent seed- and soil-borne fungal pathogens of maize. The fungus may cause severe ear rot and fumonisin mycotoxin contamination of the kernels. Alternative to its pathogenic effect, *F. verticillioides* often exists as a symptomless endophyte (Blacutt et al., 2018). Living inside maize tissues, *F. verticillioides* must cope with antimicrobial phytochemicals, such as the benzoxazinones and benzoxazolinones. *F. verticillioides* can tolerate BOA at concentrations that are inhibitory to other fungi. In fact, only a limited number of *Fusarium* species can tolerate BOA, with *F. verticillioides* and *F. subglutinans* being the most tolerant (Vilich et al., 1999; Glenn et al., 2001). BOA tolerance is due to hydrolysis of the five-membered oxazole ring of BOA and loss of the carbonyl group, followed by an additional modification yielding the non-toxic metabolite, N-(2-hydroxyphenyl) malonamic acid (HPMA) (Glenn et al., 2002; Glenn et al., 2003; Glenn and Bacon, 2009; Glenn et al., 2016). It is worth mentioning that the oxazole ring consists of moieties for both a γ-lactam and γ-lactone (**Figure 1**).

Our research group previously characterized a metallo-beta-lactamase gene (*MBL1*) in *F. verticillioides* essential for BOA tolerance, and deletion of this gene rendered *F. verticillioides* incapable of metabolizing BOA (Glenn et al., 2016; Gao et al., 2017). Its orthologs in *F. graminearum* and *F. pseudograminearum* also demonstrated indispensable roles in BOA hydrolysis (Kettle et al., 2015). Collectively, these studies represented the first functional characterization of fungal lactamases, a group of fungal enzymes reviewed and further categorized by Gao et al. (2017).

Four compounds were selected for comparative analyses of transcriptomic responses. They were BOA, 2-oxindole (OXD), 2-coumaranone (CMN), and chlorzoxazone (CZX) (**Figure 1**). OXD is an aromatic heterocyclic organic compound consisting of a six-membered benzene ring fused to a five-membered γ-lactam ring. OXD differs from BOA in that it contains only the lactam moiety. An entomopathogenic bacterium, *Xenorhabdus hominickii*, produces OXD, which suppresses host insect responses by inhibiting eicosanoid biosynthesis, and oxindole alone can inhibit the hemocytic nodule formation of *Spodoptera exigua*, an entomopathogenic nematode species, in a dose-dependent manner (Sadekuzzaman et al., 2017). In contrast to OXD, CMN contains only the lactone moiety (**Figure 1**). There have been no reports so far describing biological functions of CMN. CZX has been prescribed as a muscle relaxant to treat spasms and the resulting pain or discomfort (Dong et al., 2006). The chemical structure of CZX is the same as BOA except for the addition of a chlorine atom on the benzene ring (5-chloro-2-benzoxazolinone; **Figure 1**).

The *MBL1* lactamase gene in *F. verticillioides* was identified through transcriptomic analysis after exposure to BOA (Glenn et al., 2016). Exploring genes that are responsive to BOA, OXD, CMN, and CZX would offer further insights into correlations between hydrolytic activities with specific moieties. Additionally, other unknowns could be addressed, such as *i*) how and in what order the lactam and lactone moieties are hydrolyzed during BOA degradation and *ii*) if there are any substrate preferences for the hydrolases?

## Materials and Methods

### Culture Preparation and Reagents

Two milliliters of PDB (potato dextrose broth; Neogen Food Safety, Lansing, MI, USA) in sterile 15 mL snap-cap tubes (Falcon, Corning, NY, USA), with caps loose, were inoculated with 10^4^ FRC M-3125 wild-type *F. verticillioides* conidia. The tubes were placed on a shaker and incubated at 27°C and 250 rpm for 47 hours, after which 5 μL DMSO containing 100 μg (final concentrations of 50 μg/mL) of either BOA (Sigma-Aldrich, St. Louis, MO, USA), OXD (Sigma-Aldrich), CMN (Sigma-Aldrich), or CZX (Sigma-Aldrich) were added as xenobiotic treatments. Five microliters of DMSO alone were added to negative control samples. All culture tubes were incubated for a final one-hour induction. Three biological replicates were prepared for each treatment. After the final hour, 1 mL liquid culture from each tube was pelleted by centrifugation at 8000xg for 5 min at 4°C, resuspended in 1 mL ice cold lysis buffer, and transferred to lysing matrix D tubes (MP Biomedicals, LLC, Santa Ana, CA, USA). The tubes were homogenized with a FastPrep-24™ 5G instrument (MP Biomedicals) at 6 m/s with 2 pulses of 30 s and a 1 min intervening pause at room temperature.

### RNA Extraction, Library Preparation and Sequencing

Total RNA was extracted from the homogenized samples with a PureLink^®^ RNA Mini Kit (Thermo Fisher Scientific Inc., MA, USA) following the manufacturer’s protocol. RNA quality was checked with an Agilent 2100 Bioanalyzer (Agilent Technologies, Palo Alto, CA, USA). Sequencing libraries were constructed with an Illumina Truseq DNA LT sample prep kit (Illumina Inc., San Diego, CA, USA) following the manufacturer’s protocol. Illumina library size validation was performed using the Agilent Tapestation 2200 High Sensitivity D1000 Assay (Part No. 5067-5584, Agilent Technologies, Santa Clara, CA, USA). Prior to equimolar library pool preparation, individual libraries were assayed for concentration by an Illumina library quantification kit (Product number KK4854, Kapa Biosystems, Inc, Wilmington, MA, USA) on a qPCR instrument (LightCycler 96, Roche Applied Science, Indianapolis, IN, USA). Each pool was clustered onboard an Illumina HiSeq2500 DNA sequencer with SR Rapid v2 flowcell clustering kits (Product number GD-402-4002, Illumina, San Diego, CA, USA). Single-end 50 bp sequencing was carried out with Rapid SBS v2 (Product number FC-402-4022, Illumina) reagents. Approximately 15 million reads were collected for BOA, OXD, and CZX libraries, and over 69 million reads were collected for CMN libraries.

### Sequencing Data Analysis

The quality of sequenced reads was assessed using FastQC (Andrews, 2010). Sequencing reads were processed by Cutadapt 1.9.dev1 to remove adapters (Martin, 2011). Low-quality reads (quality score < 30 in a 4 bp sliding window) and adapter sequences were removed by the Trimmomatic 0.32 (Bolger et al., 2014) and custom scripts to remove rRNA and organellar sequences, which were obtained from National Center for Biotechnology Information (NCBI) Gene Database (https://www.ncbi.nlm.nih.gov/). Reads were mapped to the *F. verticillioides* FGSC 7600 genome by Tophat 2.0.13 (Kim et al., 2013; Ma et al., 2010), alignment sorted by Samtools 1.2 (Li et al., 2009), and read count and expression estimation obtained by HTseq 0.6.1p1 (Anders et al., 2015) and DESeq2 (Love et al., 2014). FGSC 7600 is an alternative strain designation equivalent to FRC M-3125, the wild type used in this study. Negative binomial distribution was utilized to model read counts. Log_2_ fold changes were estimated based on a generalized linear model followed by a shrinkage normalization to account for over-dispersion. Most data are presented as log_2_ fold change, but some differential expression is discussed as ratio-based fold change [log_2_ fold change = log_2_(B) – log_2_(A); fold change = B/A = 2^log2FC^]. Finally, the Wald test was used to compare two experimental groups and calculate false discovery rate corrected p-value (Love et al., 2014).

With DESeq2, if a group of samples has read counts of zero or includes an outlier, as determined by Cook’s distance, they are considered invalid for calculating a p-value (Love et al, 2014). The analysis could include these samples by lessening these restrictions, but this was not done to report a more conservative and reliable analysis. Despite this, several genes demonstrated high differences in read counts between treatments but were not given a p-value (significant or otherwise). Choosing not to ignore them, genes with high differences in FPKM (Fragments Per Kilobase of transcript per Million mapped reads) and no assigned p-value are reported in **Supplementary Table 1**.

## Results

### BOA, OXD, CMN, and CZX Elicited Differential Gene Responses in *F. verticillioides*

The raw RNA-Seq reads of the BOA, OXD, and CZX biological replicates ranged from 14.6 to 19.7 million, of which 14.5 to 19.5 million reads were mapped to the genome of *F. verticillioides* FRC M-3125 (**Supplementary Table 2**), while reads from the CMN treated replicates ranged from 69.3 to 84.0 million (Ma et al., 2010). Greater than 96% of raw reads in each library were mapped to the *F. verticillioides* reference genome (**Supplementary Table 2**).

**Figure 2:**
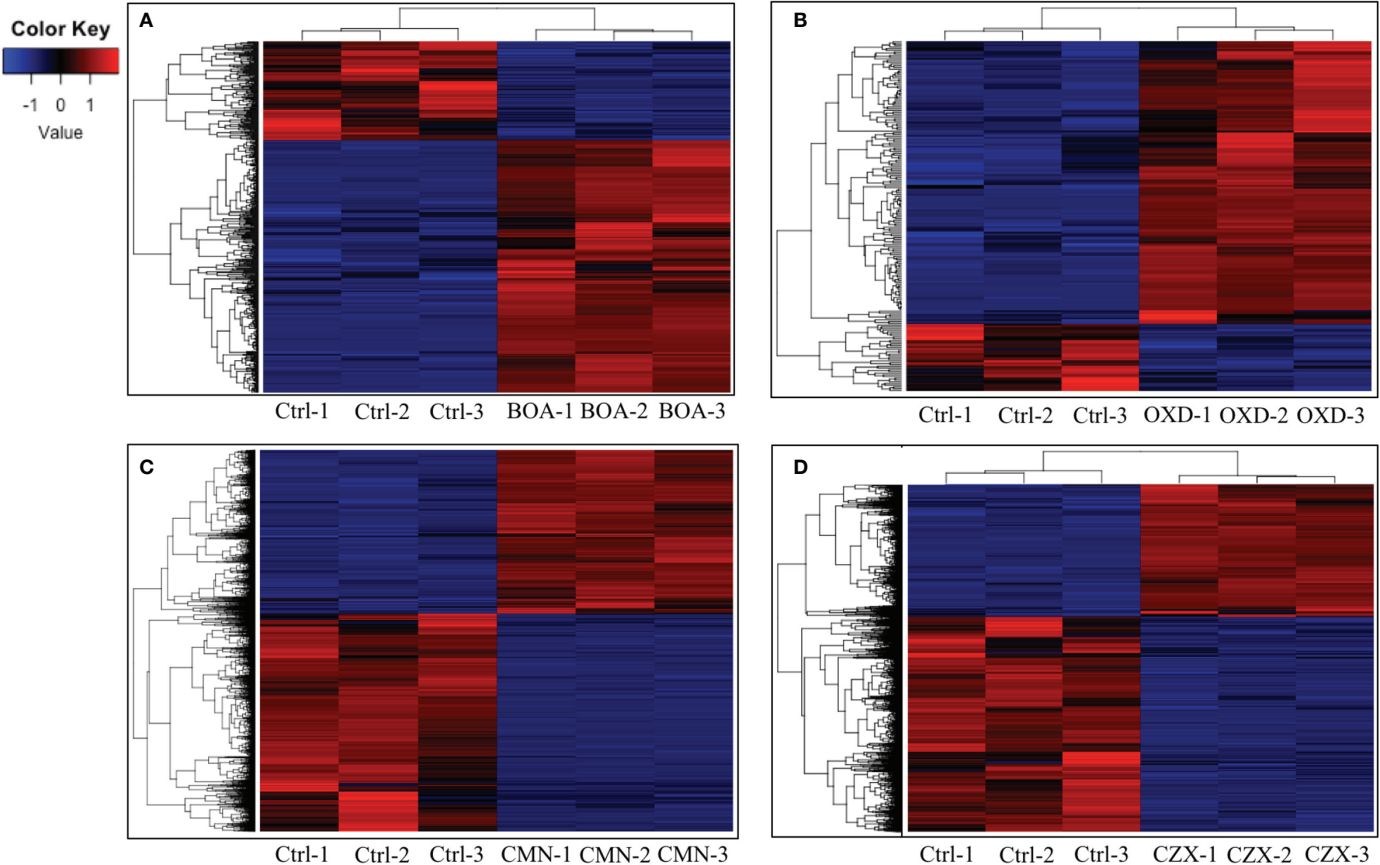
BOA, OXD, CMN, and CZX elicit differential gene expression. Heatmaps showing transcription levels of differentially expressed genes upon exposure to BOA **(A)**, OXD **(B)**, CMN **(C)**, and CZX **(D)**. The Y-axis represents genes that are clustered and colored by z-score. See the colored key. The X-axis shows the three biological replicates of each treatment.

In the BOA treatment, 587 differentially expressed genes were identified (false discovery rate adjusted p-value < 0.05, log_2_ fold change > 1 for up-regulated and log_2_ fold change < -1 for down-regulated), with 422 genes up-regulated and 165 down-regulated when compared to the DMSO control (**Figure 2A**). The down-regulated genes generally exhibited a low level of expression before BOA challenge and a minor fold change (< 6.5-fold down-regulation) after BOA challenge. RNA-Seq results corroborated previous findings (Glenn et al., 2016) that two BOA-associated gene clusters, *FDB1* on chromosome 10 (FVEG_08287–FVEG_08295) and *FDB2* on chromosome 3 (FVEG_12625–FVEG_12641), were significantly induced. Individual gene responses in both clusters ranged from no change to a 2234-fold increase following exposure to BOA (**Table 1**). The core metallo-beta-lactamase gene *MBL1* (FVEG_08291) in the *FDB1* cluster exhibited greater induction levels (761-fold) than its paralog FVEG_12637 (32-fold; FPKM = 0.36 before induction and 13 after induction) in the *FBD2* cluster, consistent with previously reported microarray data (Glenn et al., 2016).

**Table 1:** Gene clusters with differential expression upon exposure to 2-benzoxazolinone (BOA).

Gene #	log_2_(FC^a^)	p-value^b^	Annotation
FVEG_03387	6.204	6.13E-70	NAD(P)-binding Rossmann-like domain
FVEG_03388	NS^c^	NS	C2H2-type zinc finger
FVEG_03389	NS	NS	Retinal pigment epithelial membrane protein
FVEG_03390	NS	NS	Hypothetical protein
FVEG_03391	NS	NS	Cutinase transcription factor
FVEG_03392	NS	NS	Hypothetical protein
FVEG_03393	NS	NS	C6 transcription factor
FVEG_03394	2.340	2.26E-25	Fumarylacetoacetate hydrolase family
FVEG_03395	NS	NS	Cutinase
FVEG_03396	3.465	3.43E-13	Hypothetical protein
FVEG_03397	2.578	2.14E-43	Fungal specific transcription factor
FVEG_03398	1.401	3.09E-08	Secretory lipase
FVEG_03399	4.078	1.19E-34	FAD binding domain
FVEG_03400	6.794	2.77E-33	Cupin domain
FVEG_03401	NS	NS	Aldehyde dehydrogenase
FVEG_03402	2.931	1.70E-07	Sugar (and other) transporter
FVEG_03403	NS	NS	Hypothetical protein
FVEG_03404	NS	NS	Allantoate permease
FVEG_03405	1.905	0.01614446	UbiD family decarboxylases
FVEG_06020	4.636	1.07E-115	SNO glutamine amidotransferase family, PDX2
FVEG_06021	6.230	0	Pyridoxal-5’-phosphate synthase, PDX1
FVEG_06022	NS	NS	Hypothetical protein
FVEG_06023	NS	NS	Uncharacterized protein
FVEG_06024	4.747	0	1-aminocyclopropane-1-carboxylate deaminase
FVEG_08287	10.910	0	NmrA-like family
FVEG_16285	10.257	4.59E-95	Amidase
FVEG_08290	NS	NS	Dienelactone hydrolase
FVEG_08291	9.573	0	Metallo-beta-lactamase superfamily, MBL1
FVEG_08292	3.145	5.31E-75	Transmembrane amino acid transporter protein
FVEG_08293	10.909	7.63E-159	FAD binding domain
FVEG_08294	5.871	0	Fungal specific transcription factor
FVEG_08295	9.687	2.48E-105	Amidase
FVEG_08296	2.714	4.63E-39	Hypothetical protein
FVEG_09969	1.421	2.75E-05	Cytochrome P450
FVEG_09970	2.324	0.001	Amidohydrolase
FVEG_09971	NS	NS	Cytochrome P450
FVEG_09972	NS	NS	Isoflavone reductase
FVEG_16699	NS	NS	Fungal specific transcription factor
FVEG_16700	4.355	9.12E-41	ABC transporter
FVEG_09974	5.183	1.39E-98	SnoaL-like domain
FVEG_09975	5.679	1.62E-221	NAD dependent epimerase/dehydratase family
FVEG_09976	4.736	1.28E-21	Putative cyclase
FVEG_09977	5.699	5.09E-34	Transketolase, C-terminal domain
FVEG_09978	6.094	9.47E-50	Short chain dehydrogenase
FVEG_12625	9.143	3.21E-109	Dienelactone hydrolase family
FVEG_12626	NS	NS	Monocarboxylate transporter
FVEG_12627	NS	NS	Uncharacterized protein
FVEG_12628	NS	NS	3-Hydroxyacyl-CoA dehydrogenase
FVEG_12629	10.322	8.02E-141	CoA-transferase family III
FVEG_12630	4.142	1.15E-24	Salicylate hydroxylase
FVEG_17313	NS	NS	Fatty-acid amide hydrolase
FVEG_12633	9.616	0	AMP-binding enzyme
FVEG_12634	10.679	6.92E-181	Carboxylesterase family
FVEG_12635	6.069	0	Fungal specific transcription factor
FVEG_12636	9.523	0	Arylamine N-acetyltransferase, NAT1
FVEG_12637	4.989	9.67E-30	Metallo-beta-lactamase, MBL2
FVEG_17314	6.353	6.76E-26	Hypothetical protein
FVEG_12638	11.126	8.26E-206	Aldo/keto reductase family
FVEG_12639	7.908	0	Protein of unknown function
FVEG_12640	7.805	5.69E-161	Transmembrane amino acid transporter protein
FVEG_12641	9.504	0	2Fe-2S iron-sulfur cluster binding domain
FVEG_12642	7.880	7.29E-44	NADH(P)-binding
FVEG_13976	7.100	1.43E-126	L-lysine 6-monooxygenase
FVEG_13977	3.350	7.36E-131	Carboxymethylenebutenolidase
FVEG_13978	NS	NS	Activator of stress protein
FVEG_13979	7.707	9.84E-51	Short chain dehydrogenase
FVEG_13980	NS	NS	Hypothetical protein

a FC, fold change.

b False discovery rate adjusted p-value.

c NS, not significant induction.

The microarray data identified 28 genes with ≥ three-fold induction. Comparison of RNA-Seq results to the previous microarray data indicated comparable induction trends for 27 of these genes (**Supplementary Table 3**). FVEG_08792 was the exception, and we speculate differences between the microarray and RNA-Seq data may be due to differences in collection time points and processing methodologies. In addition, RNA-Seq results identified another highly up-regulated gene, FVEG_12642 (236-fold), adjacent to the *FDB2* cluster, which was not up-regulated in the previous microarray data. Unexpectedly, four new putative gene clusters were identified from the RNA-Seq results following BOA exposure, including FVEG_03387–FVEG_03405, FVEG_06020–FVEG_06024, FVEG_09969–FVEG_09978, and FVEG_13976–FVEG_13979 (**Table 1**). The induction levels ranged from 2.6- to 208-fold compared to the DMSO control. Overall, the comparison to microarray results supports the accuracy and robustness of the RNA-Seq analysis.

OXD exposure induced expression of 164 genes and down-regulated 37 genes, compared to the DMSO control (**Figure 2B**). Amongst the 164 up-regulated genes, three putative clusters were highly induced (FVEG_06533–FVEG_06536, FVEG_08763–FVEG_08774, and FVEG_13976–FVEG_13979) (**Table 2**). The induction levels ranged from 8- to 760-fold.

**Table 2:** Gene clusters with differential expression upon exposure to 2-oxindole (OXD).

Gene #	log_2_(FC^a^)	p-value^b^	Annotation
FVEG_06533	9.362	0	Amidohydrolase family protein
FVEG_06534	6.964	0	SnoaL-like domain
FVEG_06535	9.350	6.43E-80	NAD(P)-binding Rossmann-like domain
FVEG_06536	2.981	1.86E-165	Fungal specific transcription factor
FVEG_08763	4.365	1.75E-250	FAD dependent oxidoreductase
FVEG_08764	4.345	3.63E-93	Alpha/beta hydrolase family
FVEG_08765	NS^c^	NS	Hypothetic protein
FVEG_08766	NS	NS	Nitrosoguanidine resistance protein
FVEG_08767	NS	NS	Glucose 1-dehydrogenase 2
FVEG_08768	NS	NS	Cercosporin resistance protein
FVEG_08769	NS	NS	Aromatic peroxygenase
FVEG_08770	3.567	9.62E-144	Carboxypeptidase
FVEG_08771	4.330	1.36E-280	Fungal specific transcription factor
FVEG_08772	6.753	4.02E-275	Alpha/beta hydrolase family
FVEG_08773	7.248	0	CAIB/BAIF family enzyme
FVEG_08774	6.195	0	Catechol dioxygenase N terminus
FVEG_13976	9.572	5.53E-230	L-lysine 6-monooxygenase
FVEG_13977	NS	NS	Carboxymethylenebutenolidase
FVEG_13978	5.800	0	Activator of stress protein
FVEG_13979	8.955	4.08E-68	Short chain dehydrogenase
FVEG_13980	NS	NS	Hypothetical protein

a FC, fold change.

b False discovery rate adjusted p-value.

c NS, not significant induction.

Upon exposure to CMN, 4335 genes demonstrated significant differential expression compared to the control, with 1857 genes up-regulated and 2478 genes down-regulated (**Figure 2C**). Two putative gene clusters spanning genomic regions from FVEG_11952–FVEG_11959 and FVEG_12519–FVEG_12534 were significantly up-regulated (**Table 3**). The latter cluster exhibited a high level of induction and transcript abundance (FPKM increased from approximately 400 to 6000) upon exposure to CMN and contained genes putatively encoding an alpha-beta hydrolase (FVEG_12519) and a serine hydrolase (FVEG_12520) that had the greatest fold changes in the treatment, 304- and 344-fold, respectively. This cluster contains two fungal Zn(2)-Cys(6) transcription factor encoding genes (FVEG_12532 and FVEG_12534), which may be involved in cis-regulation of neighboring genes.

**Table 3:** Gene clusters with differential expression upon exposure to 2-coumaranone (CMN).

Gene #	log_2_(FC^a^)	p-value^b^	Annotation
FVEG_11952	1.325	1.28E-10	Family description
FVEG_11953	2.783	5.51E-20	Uncharacterized protein
FVEG_11954	1.570	5.05E-43	Uncharacterized protein
FVEG_11955	2.341	3.16E-124	Catalase
FVEG_11956	2.085	8.94E-90	Mo-cofactor oxidoreductase
FVEG_11957	1.782	9.35E-43	Methyltransferase domain
FVEG_11958	5.539	1.23E-46	C4-dicarboxylate transporter
FVEG_11959	3.401	7.26E-24	Uncharacterized protein
FVEG_12519	8.249	1.78E-231	Acetyltransferase
FVEG_12520	8.427	0	Serine hydrolase (FSH1)
FVEG_12521	7.539	0	Amino acid kinase
FVEG_12522	6.758	4.70E-195	YCII-related domain
FVEG_12523	7.016	0	Polyketide synthase
FVEG_12524	NS^c^	NS	Esterase
FVEG_17290	NS	NS	Acyl-CoA dehydrogenase
FVEG_17291	NS	NS	Hypothetical protein
FVEG_12526	NS	NS	Metallo-beta-lactamase
FVEG_12527	2.566	4.51E-06	Aldolase
FVEG_12528	6.750	0	Dehydrogenase
FVEG_12529	6.858	0	Sulfhydrylase
FVEG_12530	6.619	0	Dehydrogenase
FVEG_12531	5.662	0	Oxidase
FVEG_12532	2.848	1.08E-64	Fungal specific transcription factor
FVEG_12533	2.818	2.44E-84	Major facilitator superfamily
FVEG_12534	2.293	4.82E-77	Fungal specific transcription factor

a FC, fold change

b False discovery rate adjusted p-value

c NS, not significant induction

In the CZX treatment, 3331 genes were differentially expressed, including 1270 that were up-regulated and 2061 that were down-regulated (**Figure 2D**). A close inspection of fold changes and levels of significance identified two putative gene clusters, FVEG_09969–FVEG_09978 and FVEG_13976–FVEG_13979 (**Table 4**). FVEG_16700 and FVEG_09974–FVEG_09978 demonstrated highest fold changes (> 512-fold) and lowest p-values (< 1.16E-121). It is worth noting that FVEG_16700 is an ATP-binding cassette (ABC) transporter, and its adjacent putative Zn(2)-Cys(6) transcription factor FVEG_16699 was also induced (8-fold; FPKM = 98 before and 787 after induction) upon exposure to CZX. Gene FVEG_16700 also exhibited a 20-fold change in expression following BOA treatment. However, FVEG_16699 was not differentially expressed in any other treatments, although the rapid transcriptional responses of *F. verticillioides* to BOA (Glenn and Bacon, 2009) could account for a missed signal at the one-hour collection time point. To test the role of these genes in response to xenobiotics, we are developing deletion constructs for FVEG_16699, FVEG_16700, and other genes of interest. Future studies with deletion mutants will test their sensitivity to CZX and the other compounds.

**Table 4:** Gene clusters with differential expression upon exposure to chlorzoxazone (CZX).

Gene #	log_2_(FC^a^)	p-value^b^	Annotation
FVEG_09969	1.645	2.55E-08	Cytochrome P450
FVEG_09970	5.300	9.81E-22	Amidohydrolase
FVEG_09971	1.233	0.006	Cytochrome P450
FVEG_09972	NS^c^	NS	Isoflavone reductase
FVEG_16699	2.996	0	Fungal specific transcription factor
FVEG_16700	10.229	1.02E-230	ABC transporter
FVEG_09974	9.087	0	SnoaL-like domain
FVEG_09975	9.590	0	NAD dependent epimerase/dehydratase
FVEG_09976	10.893	1.16E-121	Putative cyclase
FVEG_09977	11.459	1.84E-141	Transketolase, C-terminal domain
FVEG_09978	12.179	5.34E-203	Short chain dehydrogenase
FVEG_13976	5.965	2.43E-89	L-lysine 6-monooxygenase
FVEG_13977	4.337	1.21E-219	Carboxymethylenebutenolidase
FVEG_13978	NS	NS	Activator of stress protein
FVEG_13979	7.731	1.32E-51	Short chain dehydrogenase
FVEG_13980	NS	NS	Hypothetical protein

a FC, fold change

b False discovery rate adjusted p-value

c NS, not significant induction

### Shared Induced Genes Were Observed Among Chemical Treatments

**Figure 3:**
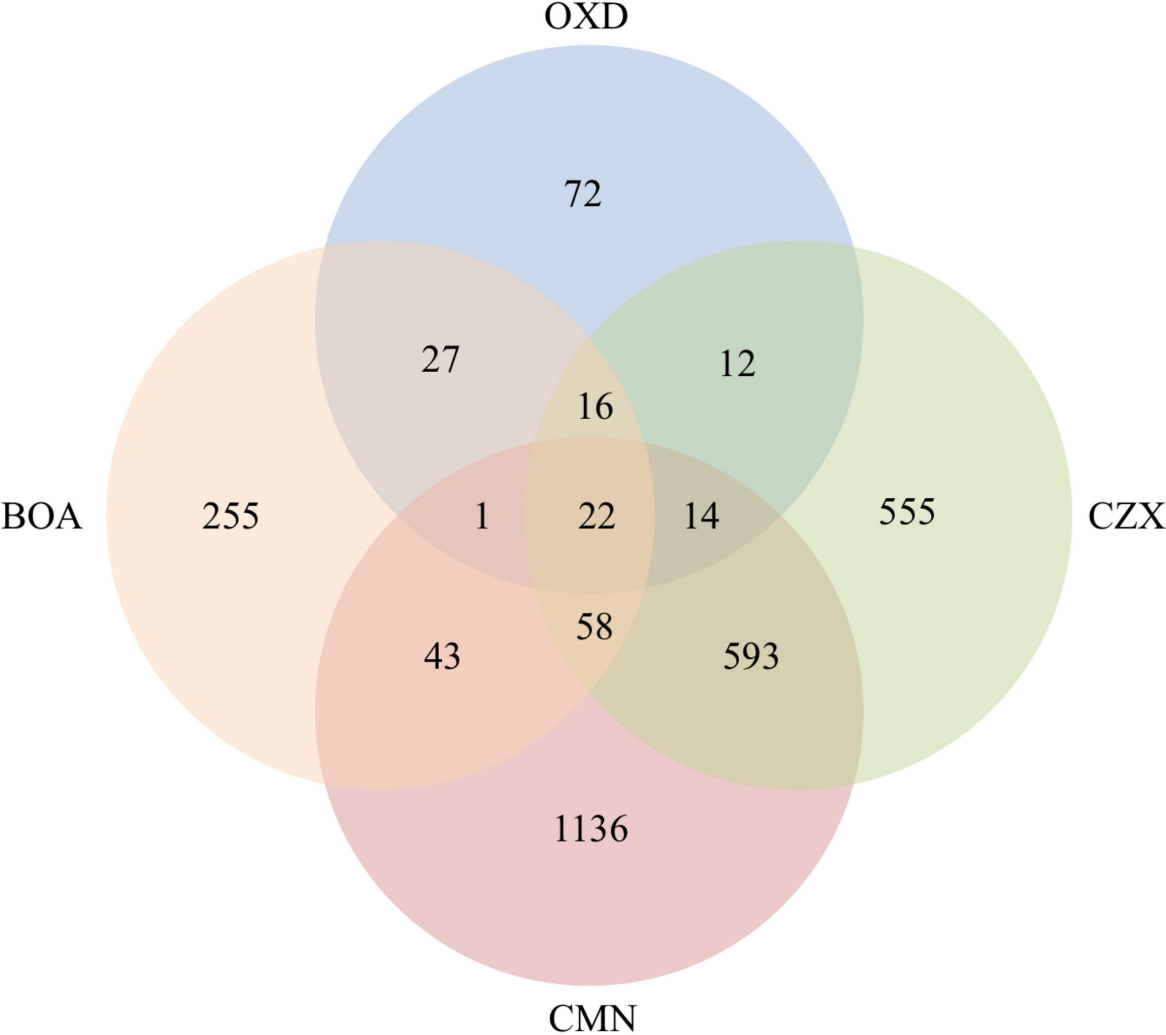
Co-upregulated genes were observed among BOA, OXD, CMN, and CZX treatments. Venn diagram shows the number of genes with altered expression due to BOA, OXD, CMN, or CZX exposure. Each circle represents one chemical treatment. Overlaps represent up-regulated genes shared between corresponding treatments.

Based on previous functional analyses of genes that were up-regulated in response to BOA, and the similarity of other chemical structures to BOA, we focused on induced genes shared among the different xenobiotics (**Figure 3**). The 22 significantly induced genes shared among all four compounds are detailed in **Table 5**. BOA and CZX contain both lactam and lactone moieties, while OXD and CMN contain only a single moiety (a lactam or a lactone, respectively). BOA, CZX, and OXD (lactam moieties) together exclusively induced 16 genes (**Figure 3**; **Table 6**). Analogously, BOA, CZX, and CMN (lactone moieties) induced a unique set of 58 genes (**Figure 3**; **Table 7**). Further, when *F. verticillioides* was treated with BOA, CZX, or CMN, genes in both the *FDB1* and *FDB2* clusters were induced. Despite induction of significant portions of the two clusters with all three treatments, BOA caused the largest number of genes to be expressed and at levels greater than CZX or CMN.

**Table 5:** Shared induced genes among BOA, OXD, CMN, and CZX treatments.

Gene #	BOA		OXD		CMN		CZX		Annotation
	log_2_(FC)^a^	p-value^b^	log_2_(FC)	p-value	log_2_(FC)	p-value	log_2_(FC)	p-value	
FVEG_00314	1.280	9.06E-03	2.048	5.85E-06	1.656	1.92E-04	1.523	2.90E-04	Zinc binding dehydrogenase, ZBD1
FVEG_01806	4.267	3.74E-197	3.318	1.61E-117	1.231	1.93E-21	1.863	4.91E-36	Thiolase, C-terminal domain
FVEG_03295	2.129	4.33E-21	1.464	2.67E-09	3.297	6.59E-62	2.397	1.66E-27	Hypothetical protein
FVEG_04214	1.183	9.83E-05	1.829	7.24E-11	2.271	5.16E-19	1.322	1.68E-06	Hypothetical protein
FVEG_05017	3.989	3.94E-211	1.747	3.79E-39	2.506	2.41E-64	3.698	7.69E-181	Coenzyme A transferase
FVEG_05278	1.401	7.17E-12	1.270	2.18E-09	1.830	6.66E-26	2.646	1.20E-42	Hypothetical protein
FVEG_06463	1.224	8.47E-05	1.019	3.08E-03	1.401	9.84E-09	3.272	5.30E-36	Aromatic ring-opening dioxygenase
FVEG_07389	5.517	4.22E-280	1.717	1.92E-25	1.869	3.26E-38	3.712	3.42E-125	Nitroreductase
FVEG_07493	2.608	6.35E-39	3.212	2.09E-59	3.186	2.44E-61	3.383	1.15E-66	NmrA-like family
FVEG_09320	1.342	4.66E-02	1.687	1.27E-02	1.684	6.28E-03	2.472	3.37E-06	Heterokaryon incompatibility protein
FVEG_09854	1.499	1.68E-18	1.337	2.69E-14	1.353	1.75E-19	1.115	3.78E-11	Beta-lactamase
FVEG_11329	1.106	9.70E-22	1.005	1.74E-17	3.161	6.18E-253	1.744	1.25E-56	Lysine N-acyltransferase
FVEG_11958	1.509	8.69E-04	1.382	4.86E-03	5.539	1.23E-46	2.054	2.54E-07	C4-dicarboxylate transporter
FVEG_12238	1.555	2.44E-26	1.003	1.23E-10	3.346	2.12E-150	2.688	2.95E-79	FAD binding domain
FVEG_12499	1.432	2.13E-24	1.701	6.39E-35	1.744	1.03E-50	2.591	1.66E-85	Methyltransferase domain
FVEG_12866	1.169	1.64E-23	1.621	4.99E-45	3.108	2.60E-225	3.515	1.87E-216	Drug resistance protein
FVEG_13057	5.150	4.07E-219	2.685	1.42E-57	2.508	3.94E-61	4.708	2.93E-182	Amidohydrolase
FVEG_13686	1.590	6.19E-36	1.571	7.88E-35	2.076	1.72E-82	3.749	7.75E-223	Methyltransferase domain
FVEG_13687	1.035	1.83E-06	1.131	2.40E-07	1.452	4.49E-17	3.235	1.61E-67	NmrA-like family
FVEG_13979	7.707	9.84E-51	8.955	4.08E-68	1.807	2.05E-03	7.731	1.32E-51	Short chain dehydrogenase
FVEG_15235	1.884	2.88E-05	1.348	1.02E-02	2.807	1.19E-11	1.889	5.84E-06	Uncharacterized protein
FVEG_16505	1.184	1.23E-03	1.091	5.97E-03	2.571	4.30E-20	1.105	1.03E-03	Uncharacterized protein

a FC, fold change.

b discovery rate adjusted p-value.

**Table 6:** Shared induced genes exclusively among BOA, OXD, and CZX treatments.

Gene #	BOA		OXD		CZX		Annotation
	log_2_(FC)^a^	p-value^b^	log_2_(FC)	p-value	log_2_(FC)	p-value	
FVEG_03387	6.204	6.13E-70	10.150	1.75E-187	7.909	4.93E-114	NAD(P)-binding Rossmann-like domain
FVEG_04841	2.522	4.86E-17	1.830	2.95E-08	2.250	6.33E-14	Hypothetical protein
FVEG_06534	1.305	3.80E-12	6.964	0.00E+00	1.035	3.57E-08	Hypothetical protein
FVEG_08763	1.885	3.45E-42	4.365	1.75E-250	1.116	3.32E-14	FAD dependent oxidoreductase
FVEG_08878	2.770	2.08E-07	9.345	6.25E-89	2.907	6.93E-09	Neutral amino acid permease
FVEG_09220	1.836	1.03E-34	1.158	6.99E-13	1.307	1.68E-17	Hemerythrin HHE cation binding domain
FVEG_09322	1.340	4.39E-08	1.183	6.46E-06	1.385	3.48E-09	Fungal specific transcription factor
FVEG_09414	4.184	2.73E-38	1.910	9.64E-08	1.318	8.22E-05	1,2-Dioxygenase
FVEG_10517	1.152	2.37E-09	1.226	4.02E-10	1.151	3.97E-10	3-Hydroxyacyl-CoA dehydrogenase
FVEG_10905	5.394	1.57E-68	6.424	3.99E-97	4.603	3.92E-50	Related to integral membrane protein PTH11
FVEG_12220	5.566	0.00E+00	5.954	0.00E+00	5.143	3.29E-278	SnoaL-like domain
FVEG_12508	2.478	1.81E-31	2.132	1.30E-22	2.797	7.27E-41	Thiamine pyrophosphate enzyme
FVEG_12509	5.420	3.83E-42	4.056	2.13E-22	3.301	3.33E-15	Acyl-CoA reductase (LuxC)
FVEG_13976	7.100	1.43E-126	9.572	5.53E-230	5.965	2.43E-89	L-lysine 6-monooxygenase
FVEG_13977	3.350	7.36E-131	5.800	0.00E+00	4.337	1.21E-219	Carboxymethylenebutenolidase

a FC, fold change.

b False discovery rate adjusted p-value.

**Table 7:** Shared induced genes exclusively among BOA, CMN, and CZX treatments.

Gene #	BOA		CMN		CZX		Annotation
	log_2_(FC)^a^	p-value^b^	log_2_(FC)	p-value	log_2_(FC)	p-value	
FVEG_00022	2.588	5.87E-33	2.094	5.00E-23	2.943	1.10E-43	Zinc-binding dehydrogenase
FVEG_00023	4.022	2.13E-104	2.753	6.87E-50	2.919	9.96E-53	Cytochrome P450
FVEG_00094	1.410	1.31E-06	2.829	1.19E-31	1.350	7.16E-07	Hypothetical protein
FVEG_00201	1.149	6.09E-04	2.848	4.54E-32	1.678	6.66E-09	ADP-ribose pyrophosphatase
FVEG_00220	1.180	2.61E-04	1.363	5.61E-08	1.124	1.47E-04	Hypothetical protein
FVEG_01737	2.439	3.04E-05	2.551	1.08E-03	1.589	6.36E-03	Flavin-binding monooxygenase-like
FVEG_01983	1.063	4.05E-17	1.836	5.91E-70	1.822	7.35E-52	Putative pantothenate kinase
FVEG_02001	1.933	4.35E-13	1.737	1.57E-11	1.683	2.22E-10	Hypothetical protein
FVEG_02121	1.428	2.88E-34	1.868	3.35E-80	2.057	3.14E-72	3,4-dihydroxy-2-butanone 4-phosphate synthase
FVEG_02122	1.073	2.52E-37	1.337	1.49E-84	1.424	8.97E-67	Ribosomal protein L11 methyltransferase
FVEG_02231	1.828	1.75E-45	1.327	2.87E-32	1.314	4.93E-24	Histidine phosphatase superfamily
FVEG_03096	1.107	3.98E-14	1.557	2.71E-39	1.044	4.42E-13	Domain of unknown function (DUF4009)
FVEG_03434	1.076	4.81E-13	1.774	2.74E-39	2.099	9.17E-53	Thioesterase-like superfamily
FVEG_03678	1.330	8.89E-26	1.048	2.44E-20	1.890	8.31E-53	Fungal specific transcription factor
FVEG_03712	1.105	2.90E-09	1.773	2.56E-27	1.092	9.10E-10	NAD(P)-binding Rossmann-like domain
FVEG_05402	1.042	4.62E-08	1.387	1.23E-21	1.189	3.77E-11	Ribosomal protein
FVEG_05486	3.159	7.60E-187	1.288	5.63E-28	1.512	1.52E-41	Flavin reductase like domain
FVEG_06021	6.230	0.00E+00	2.137	2.58E-46	2.164	3.79E-39	Pyridoxal-5’-phosphate synthase, PDX1
FVEG_06355	1.327	2.61E-23	2.119	1.86E-74	2.575	1.40E-88	PPR repeat family
FVEG_06356	1.222	8.75E-15	2.064	3.59E-55	2.438	1.06E-60	Helicase conserved C-terminal domain
FVEG_07842	1.518	2.43E-13	1.157	4.10E-10	1.667	7.58E-17	Hypothetical protein
FVEG_08287	10.910	0.00E+00	1.510	3.59E-06	1.966	4.99E-10	NmrA-like protein
FVEG_08293	10.909	7.63E-159	1.457	4.81E-04	1.836	1.76E-05	FAD binding domain
FVEG_08296	2.714	4.63E-39	1.419	1.00E-12	2.048	1.22E-21	Major facilitator superfamily
FVEG_08301	1.250	3.71E-08	1.859	6.66E-26	1.359	2.67E-10	Glycosyl hydrolases family
FVEG_08506	1.046	6.54E-14	1.665	1.18E-45	2.042	9.12E-54	Hypothetical protein
FVEG_08507	1.161	4.04E-14	2.344	3.16E-73	1.817	4.10E-36	Phospholipid methyltransferase
FVEG_09401	4.016	1.14E-24	1.770	5.11E-05	1.184	1.18E-02	Zinc-binding dehydrogenase
FVEG_09796	2.428	3.68E-96	2.055	4.18E-93	1.217	1.39E-23	Serine hydrolase (FSH1)
FVEG_09813	1.494	1.20E-46	1.957	6.60E-112	1.356	1.56E-38	Amidohydrolase
FVEG_09841	4.083	1.31E-20	4.746	3.71E-12	4.276	2.35E-23	MmgE/PrpD family
FVEG_09842	2.299	1.56E-03	4.286	1.70E-03	2.226	6.51E-04	SP: MFS transporter, sugar porter (SP) family
FVEG_09878	1.778	4.57E-03	2.215	1.30E-03	1.889	5.92E-04	Xylose isomerase-like TIM barrel
FVEG_10067	1.209	9.72E-08	1.325	1.67E-11	1.679	7.36E-16	Hypothetical protein
FVEG_10938	1.487	9.13E-25	1.912	4.83E-58	1.706	2.64E-33	FAD binding domain
FVEG_11172	1.336	6.31E-54	1.031	3.53E-44	1.693	2.23E-87	GTP cyclohydrolase II
FVEG_11330	1.194	2.39E-25	2.834	2.02E-197	2.530	8.23E-117	AMP-binding enzyme
FVEG_11398	1.118	1.10E-28	1.662	9.11E-85	1.718	2.19E-68	Adenylate kinases
FVEG_11439	4.487	2.03E-50	3.093	5.10E-21	2.082	4.97E-10	Endoribonuclease L-PSP
FVEG_11507	1.931	2.21E-04	2.922	3.53E-10	1.951	4.44E-05	Hypothetical protein
FVEG_12174	1.729	5.54E-50	1.462	8.89E-47	1.297	4.94E-27	Terpene synthase family, metal binding domain
FVEG_12625	9.143	3.21E-109	4.080	7.37E-17	3.136	9.24E-13	Dienelactone hydrolase family
FVEG_12634	10.679	6.92E-181	2.236	1.05E-08	1.941	1.63E-06	Carboxylesterase family
FVEG_12635	6.069	0.00E+00	1.733	2.13E-91	1.788	2.64E-85	Fungal specific transcription factor
FVEG_12636	9.523	0.00E+00	1.273	1.13E-16	2.430	1.55E-49	Arylamine N-acetyltransferase, NAT1
FVEG_13006	5.246	6.76E-55	1.829	3.07E-06	1.966	2.63E-07	Hypothetical protein
FVEG_13056	1.120	1.43E-06	1.783	9.47E-24	1.738	1.10E-16	Fungal specific transcription factor
FVEG_13061	1.054	2.37E-02	1.939	2.21E-07	2.181	8.83E-10	Hypothetical protein
FVEG_13807	2.339	4.63E-04	2.395	2.02E-02	1.595	1.30E-02	KR domain
FVEG_13808	1.678	4.62E-07	1.148	1.43E-04	2.263	8.37E-14	SnoaL-like domain
FVEG_13989	2.865	2.08E-18	2.147	7.72E-12	1.147	1.88E-03	SMP-30/Gluconolaconase/LRE-like region
FVEG_14011	1.620	3.82E-02	2.835	4.37E-02	2.023	1.70E-03	Alpha amylase, catalytic domain
FVEG_14160	4.321	4.01E-111	1.445	9.04E-15	1.336	4.77E-10	Glycosyl hydrolase family
FVEG_14949	1.107	3.22E-02	1.384	6.26E-04	1.175	7.59E-03	Hypothetical protein
FVEG_15086	2.421	3.35E-04	2.822	5.89E-03	1.468	2.50E-02	Hypothetical protein
FVEG_15221	1.032	4.05E-06	2.081	2.16E-32	1.623	7.52E-16	Hypothetical protein
FVEG_15302	3.254	9.58E-11	2.353	2.82E-05	3.100	1.70E-10	Short chain dehydrogenase
FVEG_16409	1.551	3.60E-02	2.193	2.91E-02	2.173	2.86E-04	Berberine and berberine like
FVEG_17021	1.282	5.36E-03	2.235	8.69E-07	1.070	9.34E-03	Hypothetical protein

a FC, fold change.

b False discovery rate adjusted p-value.

### Shared Induced Gene Clusters Were Observed Between BOA and BOA-Like Treatments

A three-gene cluster, consisting of FVEG_13976, FVEG_13977, and FVEG_13979 was highly up-regulated with BOA, CZX, and OXD treatments, ranging from 10- to 724-fold induction compared to the DMSO control (**Tables 1**, **2**, **4**, **6**). However, FVEG_13976 and FVEG_13977 were not significantly induced upon exposure to CMN. Compared to the extremely high levels of induction in BOA, CZX, and OXD treatments (> 120-fold change; FPKM > 2000 after induction), a nominal induction of FVEG_13979 was observed with the CMN treatment, an approximately 3-fold change and a FPKM less than 10 after induction.

The BOA and CZX treatments shared a highly induced 11-gene cluster with accession numbers FVEG_09969-FVEG_09978 (**Tables 1**, **4**). Genes in the cluster were up-regulated from 20- to 68-fold when *F. verticillioides* was exposed to BOA, while the level of induction was even higher in the CZX treatment, ranging from 541- to 4608-fold. In contrast, exposure to OXD or CMN did not elicit differential expression of these genes, suggesting this cluster may respond to the combination of adjacent lactam and lactone moieties in BOA and CZX.

### Differential Expression of Lactamase Encoding Genes Was Observed Across Chemical Treatments

Induction of lactamase genes upon exposure to the xenobiotics was assessed. In addition to the previously validated up-regulation of *MBL1* (FVEG_08291) and *MBL2* (FVEG_12637) in BOA treatments (Glenn et al., 2016), we also observed the significant induction of two more lactamase encoding genes, FVEG_09854 and FVEG_12526 (**Table 8**). FVEG_12347 was the only down-regulated lactamase encoding gene upon exposure to BOA (**Table 8**). Exposure to CMN caused the differential expression of ten lactamase encoding genes in total, with four genes up-regulated and six genes down-regulated (**Table 8**). Exposing *F. verticillioides* to OXD did not down-regulate any of the lactamase encoding genes, and the previously noted FVEG_09854 was the only up-regulated lactamase encoding gene with a 2.5-fold induction. CZX elicited the differential expression of eight lactamase-encoding genes. Of the four CZX up-regulated beta-lactamases (FVEG_03849, FVEG_05734, FVEG_09854, and FVEG_12457), FVEG_09854 was also induced by BOA, OXD and CMN treatments (**Table 8**). Exposure to CZX elicited an equal number of down-regulated lactamase encoding genes, and two of them (FVEG_13172 and FVEG_13253) were also repressed by the CMN treatment.

**Table 8:** Lactamase genes differentially expressed upon exposure to BOA, CMN, OXD, and CZX.

Group	Gene#	Mean FPKM		log_2_(FC)^a^	p-value^b^
		w/o Induction	w/Induction		
BOA-up	FVEG_08291	3.199	2765.573	9.573	0.000E+00
	FVEG_09854	78.535	225.985	1.499	1.680E-18
	FVEG_12526	0.114	0.607	1.704	2.069E-02
	FVEG_12637	0.359	13.138	4.989	9.670E-30
BOA-down	FVEG_12347	1.263	0.411	-1.366	1.007E-02
CMN-up	FVEG_08291	3.151	5.917	1.035	1.763E-07
	FVEG_09854	77.331	194.239	1.353	1.751E-19
	FVEG_10996	2.166	5.186	1.297	7.105E-15
	FVEG_12159	9.454	65.409	2.737	6.898E-156
CMN-down	FVEG_04555	9.133	3.657	-1.282	1.142E-17
	FVEG_05854	8.558	0.288	-4.986	2.743E-71
	FVEG_09904	4.591	1.972	-1.274	3.837E-07
	FVEG_12637	0.353	0.088	-2.039	2.123E-03
	FVEG_13172	348.896	1.585	-7.794	3.726E-220
	FVEG_13253	0.839	0.279	-1.539	2.664E-04
OXD-up	FVEG_09854	78.535	201.618	1.337	2.69E-14
CZX-up	FVEG_03849	64.211	142.193	1.141	5.046E-38
	FVEG_05734	1.310	4.433	1.690	1.324E-09
	FVEG_09854	78.535	201.619	1.337	2.686E-14
	FVEG_12457	0.455	1.620	1.608	1.114E-04
CZX-down	FVEG_09904	4.662	0.642	-2.585	1.433E-14
	FVEG_12347	1.263	0.146	-2.142	1.647E-05
	FVEG_13172	354.235	71.106	-2.205	1.548E-15
	FVEG_13253	0.851	0.091	-2.131	8.939E-05

a FC, fold change.

b False discovery rate adjusted p-value.

### MIPS Functional Categorization Revealed Differential Trends in Genes Responsive to BOA and BOA-Like Compounds

To gain insight into the functional categories of genes responsive to the four chemical treatments, MIPS FunCat system was utilized with gene lists from differential expression analyses (Ruepp et al., 2004). Functional categories enriched in response to BOA were those involved in metabolism, energy, protein degradation, cell rescue, and interaction with the environment (**Table 9**). Similarly, OXD-responsive genes were putatively involved in the majority of the above categories with one additional gene associated with signal transduction. None of the OXD-responsive genes were functionally enriched in energy metabolism. More enriched functional categories, including systemic development and biogenesis of cellular components, were observed with both CMN and CZX treatments, compared to BOA and OXD. Since there were more genes differentially expressed upon exposure to CMN and CZX than to BOA or OXD, each enriched category from the CMN and CZX treatments contained a larger number of genes than did the BOA or OXD treatments (**Table 9**). Despite the differences in the total number of induced genes among the four treatments, approximately 31% - 36% were enriched in the metabolism category. Further, CMN and CZX treatments demonstrated a significantly higher correlation between metabolism and down-regulation, compared to BOA or OXD treatments. Approximately 15 to 19% of induced genes in BOA, CMN, and CZX treatments were enriched for cellular transport, but with the OXD treatment it was less than 10%.

**Table 9:** MIPS functional categorizations for genes differentially regulated in response to BOA, OXD, CMN, and CZX.

Functional Category	BOA		OXD		CMN		CZX	
	Up	Down	Up	Down	Up	Down	Up	Down
	(422)^a^	(165)	(164)	(37)	(1867)	(2478)	(1270)	(2061)
01 Metabolism	153	29	58	16	587	582	394	503
02 Energy	30				127	51		
12 Protein synthesis								31
14 Protein fate (folding, modification, destination)				2			33	
16 Protein with binding function or cofactor requirement	28		15		139	5	268	89
20 Cellular transport, transport facilities and routes	71	39	26	3	308	361	253	297
30 Cellular communication/signal transduction				1				
32 Cell rescue, defense and virulence	52	6	33		199	216	150	216
34 Interaction with the environment	6			3	57		81	6
41 Systemic development						5		
42 Biogenesis of cellular components					35		36	

a Total number of F. verticillioides up-regulated and down-regulated genes in response to the four xenobiotics is shown in parentheses.

## Discussion

OXD, CMN, and CZX are three lactam and/or lactone containing compounds with related chemical structures to the maize phytoanticipin, BOA. Inspired by *F. verticillioides* transcriptional and functional analyses of lactamase containing gene clusters associated with BOA degradation, additional transcriptional inferences were explored by exposing *F. verticillioides* to BOA, OXD, CMN, and CZX. Several putative degradative gene clusters were elicited during chemical exposure, and these clusters were either exclusive to a particular xenobiotic or shared between various treatments.

### Putative Hydrolytic Genes and Gene Clusters Were Identified Upon Exposure to BOA

Regarding the *FDB1* and *FDB2* gene clusters, RNA-Seq results were consistent with the previous microarray data for BOA (Glenn et al., 2016). The RNA-Seq experiment further identified a larger number of genes differentially expressed by *F. verticillioides* when exposed to BOA. Such differences between the microarray and RNA-Seq datasets is not surprising given the two methodologies and the differing culture conditions for the two experiments.

Among the induced genes identified from RNA-Seq, FVEG_12642 exhibited a 236-fold induction upon exposure to BOA. This gene putatively encodes a NmrA-like protein of 301 amino acids in length, which possibly functions as a transcription regulator and NADPH sensor. FVEG_12642 is located adjacent to the previously characterized *FDB2* cluster, but it was not noted in the previous microarray data. The induction level is comparable to other induced genes in the *FDB2* cluster. Orthologs to FVEG_12642 are widely present in other *Fusarium* species with over 98% sequence identities (data not shown) as opposed to the limited identity over the entire *FDB2* cluster.

In addition to the two previously characterized BOA-induced *FDB1* and *FDB2* gene clusters (Glenn et al., 2002; Glenn and Bacon, 2009; Glenn et al., 2016), here we identified two additional gene clusters (**Table 1**). The FVEG_03387–FVEG_03405 cluster contains a fumarylacetoacetate hydrolase (FVEG_03394), which was previously studied in human clinical research for its role in tyrosine catabolism in the liver and kidney (Awata et al., 1994). Fumarylacetoacetate hydrolase is thought to be involved in the catabolism of phenylalanine, and the chemical structure of phenylalanine is reminiscent of N-(2-hydroxyphenyl)malonamic acid (HPMA), which is the metabolic product of BOA hydrolysis and malonylation (Glenn and Bacon, 2009; Glenn et al., 2016). It is possible that FVEG_03394 is regulated by a break-down product of BOA and contributes to further catabolic activity. The FVEG_03387–FVEG_03405 cluster is also located directly adjacent to the bikaverin (BIK) biosynthetic gene cluster (FVEG_03379–FVEG_03384) (Linnemannstons et al., 2002), but the genes in the BIK cluster did not demonstrate differential expression upon exposure to BOA. It may be worth pursuing the impact of the BOA-induced FVEG_03387–FVEG_03405 cluster on BIK biosynthesis.

The FVEG_06020–FVEG_06024 cluster contains the genes governing the biosynthesis of pyridoxine, also known as vitamin B6, which is an efficient singlet oxygen quencher and antioxidant contributing to reactive oxygen species (ROS) resistance (Bilski et al., 2000). For example, vitamin B6 protects *Cercospora nicotianae* from ^1^O_2_-mediated damage (Ehrenshaft et al., 1999). FVEG_06020 encodes a homolog of PDX2, a glutamine amidotransferase, and FVEG_06021 encodes a homolog of PDX1, a pyridoxal-5’-phosphate (PLP) synthase. These are the core enzymes for biosynthesis of pyridoxine (Titiz et al., 2006; Tambasco-Studart et al., 2007). Lastly, FVEG_06024 putatively encodes the PLP-dependent 1-aminocyclopropane-1-carboxylate deaminase, which hydrolyzes 1-aminocyclopropane-1-carboxylate (ACC) to ammonia and alpha-ketobutyrate (Van de Poel and van der Straeten, 2014). ACC is a precursor of ethylene, and ACC synthase is also a PLP-dependent enzyme that utilizes vitamin B6 as a co-factor for its enzymatic function (Boller et al., 1979). Thus, in addition to ROS resistance, this cluster may confer to *F. verticillioides* the ability to mediate ethylene-related hormonal activity within its primary host, maize.

### BOA and OXD Exposure Revealed Specific Clusters With Functional Similarity

Another BOA- and CZX-induced gene cluster, FVEG_09969–FVEG_09978, contained genes encoding an amidohydrolase (FVEG_09970), a SnoaL-like domain (FVEG_ 09974), a NAD-binding domain (FVEG_09975), and additional hydrolases, which may function in hydrolyzing specific chemical moieties (**Table 1**). Exposure to OXD also elicited the expression of another gene cluster encoding an amidohydrolase (FVEG_06533), a SnoaL-like domain (FVEG_06534), and a NAD-binding domain (FVEG_06535) (**Table 2**). The one-atom difference between BOA and OXD chemical structures apparently contributes to the specific induction of clusters containing genes of similar functions. Although both clusters contained three genes with similar functional annotations, the amino acid sequences of similarly annotated genes possessed low similarities (< 5%) suggesting possible convergent function (data not shown).

### CMN Exposure Induced the Fusaric Acid Biosynthetic Gene Cluster in *F. verticillioides*

As is shown in **Table 3**, exposure to CMN induced a gene cluster from FVEG_12519 to FVEG_12534. This is the fusaric acid biosynthetic gene cluster (*FUB*) (Brown et al., 2015). Deletion of a global regulator gene, *lae1*, in *F. verticillioides* resulted in a significant down-regulation of this *FUB* cluster (Butchko et al., 2012). Conversely, we observed a significant up-regulation of the same cluster upon exposure to the lactone-containing compound, CMN. Thus, exposure to CMN may impact the production of fusaric acid by *F. verticillioides*. FVEG_12526, a metallo-beta-lactamase encoding gene located within the *FUB* cluster, was neither down-regulated from the absence of *lae1* nor induced by the presence of CMN. Future analyses will investigate the impact of CMN on fusaric acid production.

### A Dehalogenase Encoding Gene May be Involved in the Removal of Chlorine From CZX

CZX exposure elicited more than 81-fold induction of FVEG_02350, which putatively encodes a dehalogenase. With CZX being the only compound of the four possessing a halogen atom in the chemical structure, we did not observe the induction of FVEG_02350 in BOA, OXD, or CMN treatments. As the functional prediction indicates, FVEG_02350 may be involved in catalyzing the removal of the chlorine atom from CZX. This will be evaluated as part of a series of future studies.

### FVEG_16700 May Possess Substrate Specificity as a Putative Membrane Transport Protein

When challenging *F. verticillioides* with BOA and CZX, we noticed a significant induction of a membrane transporter encoding gene, FVEG_16700, with 20- and 1200-fold up-regulation, respectively. The putative protein encoded by FVEG_16700 belongs to the major facilitator superfamily, which is ubiquitously present across the different kingdoms of life and transports metabolites, drugs, amino acids, etc. across membranes (Marger and Saier, 1993). The induction of FVEG_16700 was not observed in the OXD or CMN treatments, which respectively contain only a lactam or lactone bond in their chemical structures, as opposed to the presence of both moieties in BOA and CZX. This suggests the occurrence of both lactam and lactone bonds may be inductive and possibly a structural feature for targeted excretion by the transporter.

### BOA, OXD, and CZX Share a Common Induced Gene Cluster

Although specific gene clusters were induced upon each compound treatment, we observed a common gene cluster shared among BOA, OXD, and CZX treatments, composed of three genes FVEG_13976, FVEG_13977, and FVEG_13979. FVEG_13976 putatively encodes a protein containing a NAD(P)-binding Rossmann-like domain, which often contributes to substrate binding. FVEG_13977 putatively encodes carboxymethylenebutenolidase, which belongs to a family of hydrolases, specifically those potentially acting on lactone carboxylic ester bonds (Schmidt and Knackmuss, 1980). Carboxymethylenebutenolidase typically catalyzes the hydrolysis of 4-carboxymethylenebut-2-en-4-olide, breaking it down to 4-oxohex-2-enedioate (Schmidt and Knackmuss, 1980). The 4-carboxymethylenebut-2-en-4-olide contains a carboxylic ester bond that is also present in BOA, CMN, and CZX. Oddly, induction of FVEG_13977 was not observed in the CMN treatment, but induction of the gene was seen in the OXD treatment. A possible explanation is FVEG_13977 may not function in the very first step of breaking open the lactone ring but instead may target intermediate metabolites with carboxylic ester bonds. FVEG_13979 encodes a putative short chain dehydrogenase, and most of these enzymes are NAD- or NADP-dependent oxidoreductases. Perhaps future functional analyses may include cluster deletion mutants to assess changes in degradation products when exposed to the four xenobiotics.

### BOA, CMN, and CZX Cause Differences in Induction of *FDB1* and *FDB2*

While BOA caused the greatest change in expression to both *FDB1* and *FDB2* gene clusters, CMN and CZX also induced portions of the clusters. Perhaps *F. verticillioides* is more responsive to BOA, a compound the fungus routinely encounters in the cornfield environment. Such rapid and intense induction by BOA, despite the short exposure time of 1 h, may be why BOA was the only compound to cause full activation of *FDB1* and *FDB2*. An earlier study demonstrated that *NAT1*, a gene within the *FDB2* cluster encoding an arylamine *N*-acetyltransferase, can be induced with exposure to BOA in as little as 15 minutes (Glenn and Bacon, 2009). Evidence to support this may be seen in the expression of FVEG_12635, a transcription factor located within *FDB2*. In the CMN and CZX treatments, expression of FVEG_12635 was almost 4 times higher than that of the DMSO control, whereas in the BOA treatment, the expression was 64 times greater than the control, supporting the idea of this cluster being quickly induced by BOA. Similar differences were also seen in the transcription factor FVEG_08294 of the *FDB1* cluster when comparing BOA and CZX treatments. In response to CMN, FVEG_08294 did not meet our threshold for being considered induced. As these responses were not seen in the OXD treatment, it is possible that these clusters may be responding primarily to the lactone moiety within these compounds.

In summary, exposure to BOA, CMN, OXD, or CZX elicited the differential expression of genes and putative gene clusters that are either exclusive to a particular treatment or shared among them. It is likely that certain genes or gene clusters may not be induced to directly catabolize BOA, CMN, OXD, or CZX, but instead may be responding to their degradation intermediates. Further, *F. verticillioides* may be biosynthesizing compounds, such as antioxidants, in response to physiological challenges like the generation of ROS resulting from xenobiotic exposure. The induction of the putative pyridoxine biosynthetic cluster is consistent with this hypothesis. The induction of the fusaric acid gene cluster by CMN, and similarly the BOA induction of the putative cluster adjacent to the BIK biosynthetic gene cluster, suggest a link between BOA or BOA-like compound exposure and production of secondary metabolites. Plant and soil associated fungi such as *F. verticillioides* are thought to encounter a large diversity of lactam and lactone compounds in their natural environments that could impact their overall fitness, virulence, or persistence (Gao et al., 2017). Our data are informative and useful for directing future functional analyses of genes potentially involved in specifically targeting the lactam and lactone moieties of these xenobiotics.

## Conclusion

Many antibacterial and antifungal chemicals have structural features defining them as lactams or lactones. Likewise, many bacteria and fungi produce hydrolytic lactamase and lactonase enzymes for degradation of these inhibitory compounds. This study identified genetic responses of the maize fungal pathogen *F. verticillioides* to four different structurally related lactam/lactone compounds. By investigating the differentially expressed genes that are shared or unique to each compound, new detoxification strategies or antifungal compounds may be identified along with new gene targets for subsequent study.

## Data Availability Statement

The datasets presented in this study can be found at https://www.ncbi.nlm.nih.gov/geo/, GSE116351.

## Author Contributions

MG, SG, BS, and AG conceived the study and designed the experiments. MG and MD performed the experiments. MG, XG, TS, SG, and AG analyzed the data. MG, XG, TS, SG, and AG wrote the manuscript. All authors contributed to the study and approved the final version of the manuscript.

## Funding

This work was supported by U.S. Department of Agriculture, Agricultural Research Service (USDA-ARS) project number 6040-42000-043-00D.

## Conflict of Interest

Authors MG and XG are employed by Bayer R&D Services LLC.

The remaining authors declare that the research was conducted in the absence of any commercial or financial relationships that could be construed as a potential conflict of interest.

## Publisher’s Note

All claims expressed in this article are solely those of the authors and do not necessarily represent those of their affiliated organizations, or those of the publisher, the editors and the reviewers. Any product that may be evaluated in this article, or claim that may be made by its manufacturer, is not guaranteed or endorsed by the publisher.
