# Supplementary material for: Transcriptomic Responses of Fusarium verticillioides to Lactam and Lactone Xenobiotics
Source: Front Fungal Biol. 2022 Jun 20;3:923112. doi: 10.3389/ffunb.2022.923112 (PMC10512309; doi:10.3389/ffunb.2022.923112)
Supplement: Supplementary file 1 [file DataSheet_1.docx]

Supplementary Material

**Supplementary Table 1.** *Fusarium* *verticillioides* genes with high FPKM values but no significant p-value after exposure to BOA, OXD, or CZX

|  | ***Fusarium* *verticillioides* genes** | | | | | |
| --- | --- | --- | --- | --- | --- | --- |
| **Replicate treatments & log2(FC)** | **FVEG_07608** | **FVEG_08290** | **FVEG_08879** | **FVEG_12628** | **FVEG_13980** | **FVEG_14165** |
| Ctrl-1 | 0.46 | 0.75 | 1.05 | 0.00 | 0.71 | 0.69 |
| Ctrl-1 | 0.00 | 0.07 | 0.04 | 0.00 | 0.00 | 0.00 |
| Ctrl-1 | 0.00 | 0.00 | 0.05 | 0.07 | 0.09 | 0.20 |
|  |  |  |  |  |  |  |
| BOA-1 | 728.58 | 4340.35 | NS | 237.70 | 649.34 | 1402.85 |
| BOA-2 | 661.25 | 4216.18 | NS | 241.62 | 736.27 | 1375.26 |
| BOA-3 | 675.29 | 4332.31 | NS | 228.66 | 626.27 | 1406.00 |
| log_2_(FC) | 7.36 | 10.31 | NS | 8.19 | 6.60 | 8.32 |
|  |  |  |  |  |  |  |
| OXD-1 | NS | NS | 564.27 | NS | 2138.98 | NS |
| OXD-2 | NS | NS | 428.36 | NS | 2048.10 | NS |
| OXD-3 | NS | NS | 550.60 | NS | 2030.43 | NS |
| log_2_(FC) | NS | NS | 6.12 | NS | 7.97 | NS |
|  |  |  |  |  |  |  |
| CZX-1 | NS | NS | 8.51 | NS | 552.92 | NS |
| CZX-2 | NS | NS | 8.01 | NS | 431.03 | NS |
| CZX-3 | NS | NS | 8.77 | NS | 543.23 | NS |
| log_2_(FC) | NS | NS | 1.78 | NS | 6.27 | NS |

**Supplementary Table 2.** Summary of RNA-Seq mapping results

| **Replicate**  **treatments** | **Raw reads #** | **Mapped reads #** | **Mapping rates** |
| --- | --- | --- | --- |
| Control-1 | 17239023 | 17042978 | 98.90% |
| Control-2 | 17788944 | 17377585 | 97.70% |
| Control-3 | 15630242 | 15186301 | 97.20% |
| BOA-1 | 16088932 | 15906235 | 98.90% |
| BOA-2 | 14644527 | 14500927 | 99.00% |
| BOA-3 | 15013010 | 14838938 | 98.80% |
| CMN-1 | 69300901 | 68189682 | 98.40% |
| CMN-2 | 69418371 | 68145812 | 98.17% |
| CMN-3 | 83976794 | 81331723 | 96.85% |
| OXD-1 | 15672040 | 15481366 | 98.80% |
| OXD-2 | 15532169 | 15354578 | 98.90% |
| OXD-3 | 15556372 | 15400751 | 99.00% |
| CZX-1 | 19650453 | 19475090 | 99.10% |
| CZX-2 | 14598789 | 14469840 | 99.10% |
| CZX-3 | 15322677 | 15167178 | 99.00% |

Treatments are highlighted in alternate shadings.

**Supplementary Table 3.** Comparison between microarray (fold change) and RNA-Seq (log_2_ fold change) data for *F. verticillioides* genes induced by BOA

| **FVEG Locus** | **Microarray FC^a^** | **RNA-Seq log_2_(FC)^a^** |
| --- | --- | --- |
| FVEG_05718 | 7.9 | 3.6 |
| FVEG_05719 | 5.5 | 4 |
| FVEG_07606/FVEG_16149**^b^** | 3.1 | 10.1 |
| FVEG_07761 | 3.8 | 5.9 |
| FVEG_07762 | 3.2 | 3.6 |
| FVEG_08287 | 3 | 10.9 |
| FVEG_08288/FVEG_16285**^b^** | 5.2 | 10.3 |
| FVEG_08289/FVEG_16285**^b^** | 23.3 | 10.3 |
| FVEG_08290 | 37.9 | 10.3**^c^** |
| FVEG_08291 | 12.9 | 9.6 |
| FVEG_08292 | 42.6 | 3.1 |
| FVEG_08293 | 9.8 | 10.9 |
| FVEG_08294 | 4.4 | 5.9 |
| FVEG_08295 | 21.5 | 9.7 |
| FVEG_08792 | 3.3 | - |
| FVEG_09100 | 3.5 | 4.8 |
| FVEG_12625 | 4.9 | 9.1 |
| FVEG_12629 | 8.1 | 10.3 |
| FVEG_12633 | 14 | 9.6 |
| FVEG_12634 | 10 | 10.7 |
| FVEG_12635 | 5.5 | 6.1 |
| FVEG_12636 | 20.3 | 9.5 |
| FVEG_12638 | 20.4 | 11.1 |
| FVEG_12639 | 9.5 | 7.9 |
| FVEG_12640 | 8.9 | 7.8 |
| FVEG_12641 | 26.5 | 9.5 |
| FVEG_13749 | 4.5 | 5.1 |
| FVEG_14165 | 11.9 | 8.3**^c^** |
| **^a^** FC, fold change  **^b^** Genome annotations have been changed or updated since microarray study. | | |
| **^c^** Gene has > 3 log_2_ fold change in expression but lacks statistically significant p-value from DESeq2. | | |
